# Down the Iron Path: Mitochondrial Iron Homeostasis and Beyond

**DOI:** 10.3390/cells10092198

**Published:** 2021-08-25

**Authors:** Jonathan V. Dietz, Jennifer L. Fox, Oleh Khalimonchuk

**Affiliations:** 1 Department of Biochemistry, University of Nebraska, Lincoln, NE 68588, USA; jdietz2@huskers.unl.edu; 2 Department of Chemistry and Biochemistry, College of Charleston, Charleston, SC 29424, USA; foxjl@cofc.edu; 3 Nebraska Redox Biology Center, University of Nebraska, Lincoln, NE 68588, USA; 4 Fred and Pamela Buffett Cancer Center, Omaha, NE 68198, USA

**Keywords:** iron homeostasis, mitochondrial iron–sulfur clusters, heme biosynthesis, iron trafficking

## Abstract

Cellular iron homeostasis and mitochondrial iron homeostasis are interdependent. Mitochondria must import iron to form iron–sulfur clusters and heme, and to incorporate these cofactors along with iron ions into mitochondrial proteins that support essential functions, including cellular respiration. In turn, mitochondria supply the cell with heme and enable the biogenesis of cytosolic and nuclear proteins containing iron–sulfur clusters. Impairment in cellular or mitochondrial iron homeostasis is deleterious and can result in numerous human diseases. Due to its reactivity, iron is stored and trafficked through the body, intracellularly, and within mitochondria via carefully orchestrated processes. Here, we focus on describing the processes of and components involved in mitochondrial iron trafficking and storage, as well as mitochondrial iron–sulfur cluster biogenesis and heme biosynthesis. Recent findings and the most pressing topics for future research are highlighted.

## 1. Introduction

Most iron in vertebrates is used to make heme *b* cofactors for hemoglobin in red blood cells; however, the essential nature of iron derives from more than this role in oxygen transport through the bloodstream. Iron functions in a variety of cellular processes, including enzyme catalysis, DNA synthesis, gas sensing, electron transport, and mitochondrial respiration [1,2]. Iron is critical to the function of mitochondria, including the organelles’ energy-producing capabilities, which have earned them the nickname the “powerhouses of the cell” [3,4,5].

To generate ATP, mitochondria—and, more specifically, mitochondrial enzymes composing the citric acid cycle (TCA cycle) and oxidative phosphorylation system (OXPHOS)—require the iron-containing cofactors iron–sulfur clusters (ISCs) and heme [4,6,7,8]. These two types of cofactors are produced via highly conserved processes through multistep synthetic pathways that occur in part in the mitochondrial matrix [7,8]. Both ISC biogenesis and heme biosynthesis require proper iron homeostasis, which begins with environmental iron acquisition and its efficient translocation to the mitochondrial matrix.

### 1.1. Clinical Significance of Iron Homeostasis

Perturbations in iron homeostasis manifest in a vast spectrum of disorders. Oftentimes, these maladies result from mutations, which can occur in any one of a large number of genes. The most common iron-related clinical manifestations result from iron deficiency and, in the most severe cases, are diagnosed as one of numerous anemias [1,9,10]. Some iron-related diseases result from iron accumulation or overload, such as hemochromatosis and iron-loading anemias—thalassemias, congenital dyserythropoietic anemias, sideroblastic anemias, and myelodysplastic syndromes [11,12,13,14,15]. Both iron overload and deficiency are associated with adverse patient outcomes, including cardiovascular disease, cognitive impairment and neurodegeneration, organ failure, diabetes mellitus, and various cancers [16,17,18,19,20,21,22,23,24,25,26,27,28,29,30,31,32,33]. Defects in heme biosynthesis cause hereditary porphyria diseases, and mutations in many of the genes involved in ISC biosynthesis also result in human disease [34,35].

At a molecular level, disruptions to mitochondrial iron homeostasis may lead to ferroptosis—a recently identified form of non-apoptotic cell death. Ferroptosis is primarily initiated by iron-dependent lipid peroxidation, which can be caused by aberrant mitochondrial iron storage, defective ISC biogenesis, or disturbed heme homeostasis (as reviewed in [36]).

### 1.2. Iron Transport and Cellular Uptake

Due to the reactivity of iron through Fenton-chemistry-like reactions, iron metabolism and transport are meticulously orchestrated by transport molecules [37]. Iron acquired through the diet is taken up by enterocytes via the plasma-membrane-bound divalent metal transporter (DMT1) or, in the case of heme, one of the plasma membrane heme importers. Feline leukemia virus subgroup C receptor-related (FLVCR) protein 2 and heme carrier protein 1 (HCP1) have been identified as heme importers in mammals, but their roles in heme transport and heme transporting mechanisms are debated [7,38,39]. Iron not used by those enterocytes is exported into bodily fluids by the transporter ferroportin or stored in ferritin.

In vertebrates, circulating ferric iron (Fe^3+^) in the bloodstream is bound to the plasma iron-transport protein transferrin (Tf). To deliver iron to tissues, Tf binds to transferrin receptor 1 (TfR1) on the cellular membrane of the target cell. The iron-loaded Tf–TfR1 assembly is endocytosed, and iron is released into the endosome, where it is reduced to the ferrous (Fe^2+^) state [40,41,42,43]. The plasma membrane metal ion transporter ZIP14 has been shown to participate in the uptake of both transferrin-bound and non-transferrin-bound iron [44,45]. Plasma heme and hemoglobin serve as alternative iron sources, since they too can be endocytosed from blood plasma following their sequestration by hemopexin and haptoglobin. From the endosome, iron relocates to the cytosol via DMT1 [46].

In unicellular eukaryotes such as the budding yeast *Saccharomyces cerevisiae*, iron is imported through several pathways. Iron import can be mediated by the Fet3/Ftr1 complex on the plasma membrane, which functions with high affinity for iron [47,48], or by a low-affinity iron import pathway, which involves the plasma membrane proteins Smf1 and Fet4 [49,50]. Similar to some prokaryotes, budding yeast can also take up iron that is siderophore-bound, in a process mediated by the ARN family transporters [51]. Unlike in metazoans, exogenous heme uptake pathways in *S. cerevisiae* are not efficient, so heme supplies a negligible amount of iron. Once within the cell, iron is stored in the vacuole until needed in the cytosol or other cellular compartments [52].

Iron in the cytosol forms the dynamic reservoir known as the cytosolic labile iron pool (CLIP). The CLIP consists of ferrous iron either stored within the protein ferritin (particularly in the liver in mammals) or chelated by low-molecular-mass ligands to form complexes. In *S. cerevisiae* and higher order eukaryotes, small molecules such as citrate, cysteine, and reduced glutathione have been proposed to serve as these ligands. To date, glutathione appears to be a strong candidate as an iron ligand, based on in vitro studies and studies in *S. cerevisiae* [53]. Iron from the CLIP can be utilized in the cytosol to metalate proteins or form ISCs, but most iron is directed to mitochondria [10,54,55].

For further discussion of cellular iron uptake and distribution, readers are referred to comprehensive reviews on this topic [56,57,58,59,60,61,62].

## 2. Mitochondrial Iron Trafficking

From the CLIP or from storage in either vacuoles (*S. cerevisiae*) or ferritin (mammals), iron is readily available to each compartment of the cell, with mitochondria being among the primary targets. In some cases, mitochondria contain more iron than the cytosol [63,64], and this iron is found within mitochondrial proteins as metal ions, ISCs, and different types of heme cofactors. Mitochondrial iron in heme and ISCs is essential for the organelles’ energy production, as it is required for electron-transfer flavoproteins, NADH:ubiquinone oxidoreductase (respiratory complex I of the ETC), succinate:ubiquinone oxidoreductase (respiratory complex II of the ETC), ubiquinol:cytochrome *c* oxidoreductase (respiratory complex III of the ETC), cytochrome *c*, cytochrome *c* oxidase (respiratory complex IV of the ETC), aconitase, lipoic acid synthase, and other proteins [10]. Mitochondria also have several diiron monooxygenases and dioxygenases involved in mitochondrial tRNA modification and cell necrosis regulation [65,66].

As with the cellular import and movement of iron, mitochondrial iron import and transport are highly regulated, meticulous processes designed to limit unwanted reactivity of the metal ions. In mitochondria, iron import and utilization involve several conserved and clinically relevant factors facilitating the efficient targeting of iron for ISC biogenesis, heme biosynthesis, and metalation of other iron-containing mitochondrial proteins (Table 1), as discussed below. For further discussion of non-mitochondrial iron metabolism, readers are referred to reviews on that topic [60,67,68,69].

### 2.1. Iron Delivery to Mitochondria

**Figure 1:**
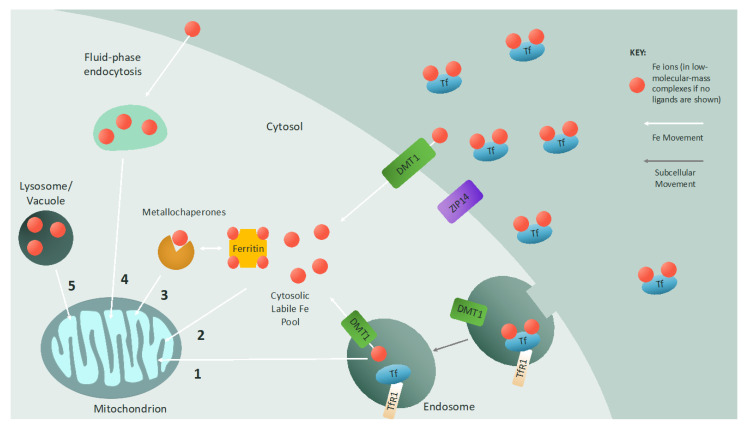
Iron delivery to the mitochondrion: Iron is imported into cells by either endocytosis (transferrin-bound iron) or plasma membrane channels (non-transferrin-bound iron). The metal transporter ZIP14 has been shown to aid in the cellular uptake of both transferrin-bound and non-transferrin-bound iron. When iron-loaded transferrin binds to the transferrin receptor on the cell surface, the protein–receptor complex is endocytosed, and iron is released into the endosome and reduced. This iron can be exported via the endosomal divalent metal transporter (DMT1) to the cytosolic labile iron pool, which consists of iron ions in low-molecular-mass complexes or stored in ferritin. Alternatively, if the endosome contacts the mitochondrion, iron is transferred to the mitochondrion via a “kiss and run” mechanism (1). Non-transferrin-bound iron imported by DMT1 on the cell membrane directly enters the cytosolic labile iron pool as iron chelated by low-molecular-mass molecules or ferritin and can then be imported into mitochondria (2). Metallochaperones such as glutaredoxin can also deliver iron to mitochondria (3). Fluid-phase endocytosis is an additional mechanism to deliver non-transferrin-bound iron to mitochondria (4). In some cases, interactions between mitochondria and lysosomes or vacuoles mediate iron transfer between organelles (5), and there may be a synergistic effect between lysosome/vacuole homeostasis and mitochondrial iron homeostasis. (Tf = transferrin; TfR1 = transferrin receptor 1).

Mitochondrial function is entirely dependent on proper iron homeostasis, and in turn, mitochondria are imperative for proper cellular iron homeostasis. Even with the importance of this mitochondria–iron relationship and the large body of research on this topic, the delivery of iron to mitochondria remains only partially understood. In mammalian cells, there is evidence for several paths of mitochondrial iron import, including endosome–mitochondria “kiss and run”, fluid-phase endocytosis, interorganellar contact sites with the lysosome, transport from the CLIP, and metallochaperone transport (Figure 1).

One pathway for the delivery of iron to mitochondria that has been studied extensively is the endosome–mitochondria “kiss and run” interaction. Primarily found in developing erythrocytes, but also demonstrated in epithelial cells, this method utilizes a direct, transient, interorganellar interaction between iron-loaded Tf-containing endosomes and mitochondria. The direct and transient nature of this interaction provides mitochondria with the excessive amounts of iron required for hemoglobin and heme synthesis during erythropoiesis [133,134,135]. This direct transfer of iron from one organelle to another bypasses the cytosolic diffusion of iron, preventing the production of reactive oxygen species via Fenton chemistry.

Another method of iron transfer to mitochondria that bypasses the cytosol and the CLIP is fluid-phase endocytosis. This pathway, proposed to occur in cardiac cells, occludes solvents from iron to directly deliver non-Tf-bound iron to mitochondria. The limited effect of chelating cytosolic iron on mitochondrial iron uptake supports this fluid-phase endocytosis model [136,137,138].

The lysosome (and lysosome-like vacuole in yeast) has recently been implicated in mitochondrial iron import and homeostasis. In erythrocytes, it appears that iron is directly conveyed to mitochondria at lysosome–mitochondria membrane contact sites to promote heme biosynthesis during erythropoiesis [139]. In yeast and mammalian cell cultures, disruptions to vacuolar and lysosomal homeostasis via an increase in alkalinity impair mitochondrial iron uptake, solidifying the roles of the lysosome and the vacuole in mitochondrial iron import and homeostasis [140,141,142,143].

Mitochondria also obtain iron from the CLIP. Low-molecular-mass iron complexes such as Fe(ATP)_2_, iron–citrate, iron–glutathione, and iron chelated by the mammalian siderophore 2,5-dihydroxybenzoic acid [144,145,146] may be imported into mitochondria, although the impact of glutathione and 2,5-dihydroxybenzoic acid on mitochondrial iron import has been contested [75]. The process of transferring iron from the CLIP to mitochondria is dependent on the mitochondrial membrane potential [147,148].

Iron trafficking is proposed to involve the coordination of iron to chaperone proteins. This mode of transportation is not unprecedented, since metallochaperones for copper (CCS and Atox1) have long been identified [149], as have several iron-specific metallochaperones. Two classes of chaperones—poly(rC)-binding proteins (specifically, PCBP1 and PCBP2) and monothiol glutaredoxins—which have been extensively studied for their roles outside of iron metabolism, have recently been the focus of iron trafficking research. PCBP1 and PCBP2 were initially identified as RNA-binding proteins required for splicing, transcript stabilization, and translational regulation. More recently, they have been shown to act as iron chaperones to deliver iron to ferritin, and for metalation of cytosolic proteins [150,151]. Glutaredoxins are glutathione (GSH)-dependent redox enzymes necessary for DNA synthesis, protein folding, signal transduction, and reactive oxygen species defense. In yeast, deletion of glutaredoxin 3 and glutaredoxin 4 results in mitochondrial iron deficiencies and impairments in both ISC and heme biogenesis. Additionally, the interaction between glutaredoxin and bovine lymphocyte antigen-like protein is required for iron delivery to mitochondria [152,153].

### 2.2. Iron Transport at the Outer Mitochondrial Membrane

Once iron is delivered to mitochondria by one of the aforementioned pathways, it must cross both the outer (OMM) and inner (IMM) mitochondrial membranes. The exact mechanism of iron transport across the OMM remains undetermined, but some factors have been speculated. Voltage-dependent anion channels (VDACs) are widely hypothesized as mitochondrial iron channels throughout eukaryotes; however, their proposed role in mitochondrial iron import relies entirely on their prevalence, involvement in other energy-related metabolite channeling, and role in the transport of other divalent cations [154]. Recent studies show that mitochondrial isoforms of DMT1 facilitate iron influx to mitochondria in several cell types, expanding the role of DMT1 in iron homeostasis beyond enterocyte iron uptake and endosomal iron export [155,156]. The aforementioned iron chaperone PCBP2, which interacts with DMT1 and with ferritin, may transport iron to other cellular compartments, such as the mitochondrion [151].

### 2.3. Iron Transport at the Inner Mitochondrial Membrane

**Figure 2:**
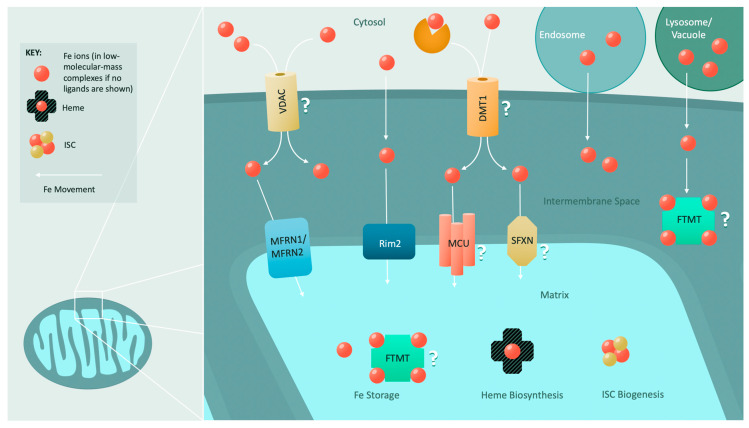
Mitochondrial iron import and utilization: The mechanism for iron import through the outer mitochondrial membrane is poorly understood. However, it is speculated that ion channels such as VDACs and a recently identified mitochondrial divalent metal transporter (DMT1) facilitate the import of iron. Interorganellar contacts with endosomes, lysosomes, and vacuoles can also convey iron to mitochondria. Transport across the inner mitochondrial membrane is primarily driven by the conserved mitoferrins (MFRN1 and MFRN2). In yeast, a low-affinity iron transporter (Rim2) assists the mitoferrins. Roles in inner mitochondrial membrane iron import have also been hypothesized for the mitochondrial calcium uniporter (MCU) and several members of the sideroflexin (SFXN) family. Once iron is imported into the mitochondrion, it is utilized for heme biosynthesis or ISC biogenesis, or it is stored in mitochondrial ferritin (FTMT) or within low-molecular-mass complexes.

Unlike iron import across the OMM, iron import at the IMM is relatively well studied (Figure 2).

Iron transport across the IMM into the matrix is dependent on proper mitochondrial membrane potential [147]. Initial discoveries of the mechanism of iron transport across the IMM were completed in yeast, where the mitochondrial solute carrier proteins—mitoferrins Mrs3 and Mrs4—are directly involved in mitochondrial IMM and matrix iron supply [157,158]. Mrs3 and Mrs4 are highly conserved from fungi to mammals and are members of a family of proteins required for IMM metabolite transport [159,160,161,162,163,164,165,166,167,168]. Mitochondrial iron levels and heme and ISC biogenesis are directly dependent on Mrs3 and Mrs4 expression [157,158,169,170]. Furthermore, genetic expression of Mrs4 is dependent on iron concentration and ISC synthesis [171,172]. However, the mechanism by which these two proteins transport iron across the IMM is presently unknown. Interestingly, it has been posited that Mrs3 and Mrs4 may also transport Cu^2+^ into the mitochondrial matrix [173,174].

Mrs3 and Mrs4 are nonessential proteins, with their deletion only being lethal in low-iron conditions, suggesting that compensatory iron uptake mechanisms must exist for the IMM [157,175]. Genetic screens in yeast mutants with defective mitochondrial iron homeostasis identified the known pyrimidine transporter Rim2 as a low-affinity mitochondrial iron transporter [175,176,177]. Overexpression of Rim2 recovers heme and ISC biosynthesis deficiencies and growth defects exhibited in *mrs3*∆ *mrs4*∆ double mutants [175,177,178,179]. Whether Rim2 follows the trend of other mitochondrial carrier proteins by co-transporting substrates remains to be determined [175,177,180,181]. Deletion of *RIM2* minimally affects mitochondrial iron homeostasis, but deletion of *RIM2* in parallel with *MRS3* and *MRS4* deletion exacerbates ISC maturation defects [176,177]. Additional genetic screens were performed to identify other IMM iron transporters; however, no candidates suppressed the phenotypes of Mrs3-/Mrs4-deficient yeast like Rim2 does; thus, it was postulated that low levels of iron nonspecifically enter mitochondria, allowing triple-deletion mutants to survive [182].

In mammalian cells, the proteins mitoferrin 1 (MFRN1) and mitoferrin 2 (MFRN2), which are functionally complementary to and homologous to Mrs3 and Mrs4 (~35% identity with Mrs4) [52,160], are responsible for iron transport across the IMM [164]. MFRN1 is the primary IMM iron transporter in differentiating erythrocytes, partly due to its extended half-life [164]. Clinically, the loss of MFRN1 causes erythropoietic protoporphyria due to defects in heme biosynthesis [160,183,184]. While the exact mechanism it employs to transport iron across the IMM remains elusive, MFRN1 is stabilized by an interaction with the IMM ATP-binding cassette (ABC) transporter ABCB10, which is likewise highly expressed in erythroid cells. This interaction also enhances mitochondrial iron import [185]. These two proteins are associated with the final heme *b* biosynthetic enzyme ferrochelatase (FECH), likely bolstering heme synthesis during erythropoiesis by stabilizing MFRN1 and funneling iron directly to FECH for integration into protoporphyrin IX to produce heme *b* [186]. Interestingly, in hepatocytes, ABCB10 has a very different role as a mitochondrial biliverdin exporter to promote the regeneration of the antioxidant bilirubin [187].

In contrast to MFRN1, MFRN2 is expressed in all vertebrate tissues (with the highest expression in the heart, skeletal muscle, liver, and kidney) and is the primary IMM iron importer of non-erythroid cells [52,160]. MFRN2 levels are increased in murine models for Friedreich’s ataxia—the iron dysregulation disease associated with mutations in the mitochondrial protein frataxin (FXN)—and in patients with muscle-specific mitochondrial iron accumulation in ISC-deficiency-related human diseases [188,189,190,191,192,193].

In conjunction with the mitoferrins, the mitochondrial calcium uniporter (MCU) may play a role in IMM iron transport as well. Several studies have provided evidence that the inhibition of MCU-mediated calcium entry prevents mitochondrial iron uptake [32,148,194].

Another group of mitochondrial proteins required for proper mitochondrial iron homeostasis has recently been uncovered: the sideroflexins (SFXN). This family of five IMM proteins is hypothesized to act either as channels or as carrier proteins [195,196]. Reduced sideroflexin expression (of SFXN4) or loss of sideroflexin function (of SFXN1) results in impaired erythropoiesis [73,196], but the precise roles of the sideroflexins in erythropoiesis remain to be determined. While SFXN1 was initially identified as a serine transporter, further analyses have revealed that it also participates in mitochondrial respiratory complex III biogenesis, activity, and assembly [197,198]. SFXN4 deficiency impairs respiration in zebrafish, and its role has been predicted to be related to mitochondrial respiratory complex I assembly, with recent studies suggesting that it is required for ISC biogenesis [73,199].

## 3. Sites of Mitochondrial Iron Utilization

Upon import into the mitochondrion, there are three primary destinations for iron: (1) iron storage, (2) ISC biogenesis and incorporation into ISC proteins, and (3) heme biosynthesis and use to form hemoproteins.

### 3.1. Mitochondrial Iron Storage

Iron can be stored in mitochondrial ferritin (FTMT)—a ferritin variant with high sequence homology to the heavy chain ferritin subunit H-ferritin [200]. In humans, FTMT is primarily expressed in the testes, and in sideroblasts of patients with sideroblastic anemia, but immunohistochemical experiments have identified FTMT in the heart, spinal cord, kidney, pancreas, and smooth muscle [200,201,202,203]. Similar to H-ferritin, FTMT also has ferroxidase activity, but this occurs much more slowly than in its cytosolic paralog [204]. The expression of FTMT significantly impacts cellular iron homeostasis; increased FTMT expression correlates with increased iron localization to mitochondria, causing mitochondrial iron accumulation [64,205]. Iron sequestration by FTMT appears to be protective against oxidative stress, since it protects mitochondria from hydrogen peroxide, antimycin A, and glucose-free conditions [206]. Further experiments are needed to determine the localization, mechanism of action, regulation, and role of FTMT in iron homeostasis.

In addition to storage in FTMT, iron can be sequestered in the mitochondrial matrix within low-molecular-mass complexes to form the mitochondrial labile iron pool [207]. GSH might serve as a ligand for mitochondrial iron since it is highly concentrated in mitochondria, is capable of chelating cytosolic ferrous iron, and has been proposed to coordinate 2Fe-2S clusters within mitochondria [208,209,210,211,212]. Citrate—another highly concentrated metabolite in the mitochondrial matrix—has also been hypothesized to function in mitochondrial iron coordination and sequestration [209,213].

### 3.2. Mitochondrial Iron–Sulfur Cluster Biogenesis and Transport

In eukaryotes, mitochondria are essential for the synthesis of all cellular ISC-containing proteins and are the site for the biogenesis of ISCs needed for mitochondrial proteins. Composed of iron and sulfide ions, ISCs are commonly found with rhomboid (2Fe-2S) or cubane (4Fe-4S) (and more rarely cuboidal, 3Fe-4S) geometries within ISC-containing proteins, where the iron atoms are ligated to cysteine thiolates or nitrogen atoms from residues such as histidine. The formation of this cofactor is critical for mitochondrial and cellular homeostasis. ISCs are involved in electron transport (e.g., ISC-containing subunits of the mitochondrial respiratory complexes I, II, and III), substrate binding and enzymatic catalysis (e.g., aconitase), nucleic acid processing and repair (e.g., ISC-containing enzymes required for DNA replication and mRNA translation), and metabolite sensing (e.g., cytosolic regulation of iron metabolism) [62,214,215,216,217,218,219,220].

The formation of ISCs in eukaryotes occurs via two pathways: one responsible for forming mitochondrial ISCs and the other responsible for forming ISCs in the cytosol. Both pathways are necessary for overall cellular health. Mitochondrial ISC biogenesis is discussed below, while the cytosolic ISC assembly (CIA) biogenesis pathway is discussed elsewhere [221,222,223].

Mitochondrial ISC biogenesis entails synthesis of the ISC on a scaffold composed of assembly proteins followed by transfer of the ISC to target apoproteins [224]. The ISC assembly factor cysteine desulfurase (NFS1) abstracts sulfur from free cysteine in a pyridoxal phosphate-dependent process that is activated by the binding of the protein ISD11 [76,225,226,227]. This sulfur, held on a catalytic cysteine as a persulfide, is transferred to the ISC scaffold protein ISCU, which directly interacts with NFS1, ISD11, and the mitochondrial acyl carrier protein (ACP1) to form the ISC biogenesis scaffold [228,229]. Electrons for the persulfide reduction, permitting formation of the ISC, are supplied by ferredoxin (which is, in turn, reduced by ferredoxin reductase) [230,231,232], while iron is supplied via an unknown mechanism [221,233]. The molecular mechanism of ISC formation remains debated, but recent work suggests the mechanism may require iron to bind to the scaffold before the sulfur is supplied [234,235].

The most probable sources of the necessary iron are the mitochondrial labile iron pool and frataxin, which can bind iron through acidic regions within the protein. Frataxin interacts with the ISCU–NSF1–ISD11 complex, and has been hypothesized to mediate iron’s entry into the ISC biogenesis scaffold and/or act as a modulator for NFS1 that might facilitate persulfide transfer to ISCU [208,221,236,237,238,239,240,241,242,243]. Decreased expression of frataxin impairs ISC biogenesis and iron metabolism, as seen in Friedreich’s ataxia [77,244].

Studies in yeast and bacteria have suggested additional roles for frataxin, including as an iron chaperone, an iron sensor and allosteric regulator of ISC biogenesis, or a metabolic switch between ISC and heme synthesis. As an iron sensor for ISC biogenesis, frataxin acts as either a negative or positive regulator of ISC biogenesis, depending on the organism. Iron binding to frataxin in bacteria decreases the rate of ISC formation, while eukaryotic frataxin enhances ISC formation via several mechanisms [238,245,246,247,248,249,250]. In eukaryotes, frataxin also interacts with ferrochelatase and regulates heme biosynthesis [251,252]. Evidence that frataxin may be acting as a metabolic switch between the formation of ISCs and heme cofactors includes its role in modulating both heme synthesis and ISC biogenesis, its ability to bind to ferrochelatase and the yeast homolog of ISCU with significantly different affinities, and its regulation by levels of protoporphyrin IX—the precursor to heme *b* [56].

Following the coordinated assembly of the ISC, the cofactor is transferred from the ISC biogenesis scaffold to target apoproteins via a mechanism that entails a chaperone–co-chaperone system. In higher eukaryotes, a member of the mitochondrial HSP70 family (HSPA9), its J-type co-chaperone HSC20, and the glutaredoxin GLRX5 mediate the transfer of ISCs to their targets. As an ATPase, HSPA9 utilizes the energy from ATP hydrolysis to induce conformational changes in the ISC-loaded ISCU, allowing ISC release from ISCU [214,253,254]. The ISC is transferred to GLRX5 which, in turn, transfers it to a recipient apoprotein, though in some cases apoproteins may receive ISCs without the intervention of GLRX5 [8,255]. Nucleotide exchange at the active site of HSPA9 is facilitated by GRPEL1 to permit future catalytic cycles [256].

How the HSPA9–HSC20 system properly determines the targets for ISC delivery remains to be determined. In some cases, a conserved leucine–tyrosine–arginine (LYR) motif in the recipient apoprotein or an accessory protein is recognized by HSC20 prior to ISC loading. This appears to be the case for many of the ISC-containing electron transport chain components [214,255,257,258]. The role of HSAP9 in ISC transport is still debated, but HSC20 has been shown to directly interact with ISCU and FXN. Depletion of this protein induces severe ISC deficits in both mitochondria and the cytosol [254,259].

In addition to their use in the maturation of 2Fe-2S cluster-containing proteins, 2Fe-2S clusters can be used to produce 4Fe-4S clusters. A dedicated complex composed of ISCA1, ISCA2, and IBA57 is required to fuse two 2Fe-2S clusters delivered by GLRX5 to form a 4Fe-4S cluster in a process that requires ferredoxin and its reductase [260,261,262,263,264]. The proteins BOLA3, NFU1, and NUBPL have all been identified as ISC targeting factors that assist in loading 4Fe-4S clusters into particular mitochondrial proteins [102,115,256,257,263,265,266,267]. GLRX5 physically interacts with BOLA3 and BOLA1, and the yeast homologs of both these BOLA family proteins have been shown to play a role in the maturation of 4Fe-4S cluster proteins [268,269].

In addition to producing ISCs for mitochondrial proteins, this mitochondrial ISC biogenesis pathway directly enables its cytosolic counterpart—the CIA—which is required to produce cytosolic and nuclear ISCs. The mitochondrial ISC pathway does this by donating a sulfur-containing (and perhaps iron-containing) intermediate from the mitochondrial matrix to the cytosol [270]. Although the exact mechanism for the transport of this intermediate is not fully understood, several studies in yeast have provided insight into the factors involved. The ABC transporter Atm1 is postulated as the IMM transporter of the sulfur-containing intermediate, and the intermembrane space (IMS) sulfhydryl oxidase Erv1 facilitates Atm1-mediated transport [271,272]. In Atm1-deficient yeast, cytosolic ISC biogenesis is disrupted, iron accumulates in mitochondria, and cellular iron uptake is enhanced [270,273]. While the involvement of the human ortholog of Atm1 (ABCB7) in transporting the intermediate to the cytosol remains to be clarified, ABCB7 has been implicated in several iron-related disorders, such as X-linked sideroblastic anemia and ataxia [272,274,275]. ABCB7 appears to be assisted in transporting the ISC intermediate by another ABC transporter (ABCB8), though the exact nature of its involvement is presently unknown. Deletion of ABCB8 in murine hearts severely impairs cardiac function due to significant mitochondrial damage, mitochondrial iron accumulation, and cytosolic ISC and heme deficiencies, which is a phenotype similar to that caused by the absence of ABCB7 [16,274,276,277]. These ABC transporters share similarity with the yeast mitochondrial peptide exporter Mdl1. Overexpression of Mdl1 partially rescues the phenotype of Atm1 deletion but, overall, Mdl1 is not implicated in iron homeostasis [278,279,280]. These data suggest that ABCB8, alongside ABCB7, may be mediating the transfer of a sulfur-containing intermediate for cytosolic ISC biogenesis.

### 3.3. Heme Biosynthesis and Transport

Heme *b*, which is an iron ion ligated by the protoporphyrin IX tetrapyrrole, and its biosynthetic derivatives heme *c* and heme *a*, are the other vital iron-containing cofactors produced by mitochondria and necessitating mitochondrial iron import. Heme is required for a plethora of processes, including oxygen transport and storage (e.g., hemoglobin and myoglobin), electron transfer (e.g., cytochrome *c*, respiratory complex III, and respiratory complex IV), signal transduction (e.g., nitric oxide synthase), ligand binding (e.g., horseradish peroxidase), transcriptional regulation (e.g., Rev-erbβ), and xenobiotic detoxification (e.g., cytochrome P450) [281,282].

The biosynthesis of heme *b* differs greatly from ISC biogenesis. In ISC biogenesis, iron is present throughout much of the process, whereas in heme biosynthesis, iron is only utilized in the final step, wherein it is incorporated into protoporphyrin IX by ferrochelatase. The process of heme biosynthesis has been studied extensively, and its details are beyond the scope of the present review; the reader is directed to reviews focused on this topic [7,283,284,285,286]. Briefly, heme biosynthesis begins with the condensation of succinyl–CoA and glycine by aminolevulinic acid (ALA) synthase to produce ALA in the mitochondrial matrix. ALA is exported to the cytosol and sequentially modified to create a cytosolic porphyrin. This porphyrin (coproporphyrinogen III) is transported back into the mitochondrion, where it is converted to protoporphyrin IX. Protoporphyrin IX and ferrous iron are combined by ferrochelatase to produce heme *b* in the mitochondrial matrix [7]. Iron is proposed to be delivered directly to ferrochelatase via protein–protein interactions with the mitoferrins [94,95,97,106,107,123]. Several enzymes in the heme biosynthetic pathway have recently been shown to interact with one another and with ABCB7, ABCB10, the protoporphyrinogen transporter TMEM14c, and the transferrin receptor to form a complex termed the heme metabolon [287].

Once synthesized, heme *b* is used to form hemoproteins required for various functions throughout mitochondria and other cellular compartments. The mechanism for the trafficking of heme to these other locales remains unknown and is the subject of ongoing research. Recent studies using ratiometric heme sensors to study heme distribution kinetics between cellular compartments in yeast revealed that the mitochondrial GTPases Gem1, Mgm1, and Dnm1 modulate heme transport to the nucleus by regulating mitochondrial architecture and endoplasmic reticulum–mitochondria contact sites [288].

Heme *b* destined for use within mitochondria is either added non-covalently to form hemoproteins or covalently modified to produce heme *c* or heme *a* for use in the hemoproteins of the electron transport chain. Heme *b* is utilized by several mitochondria-localized proteins, including the matrix protein neuroglobin (flavohemoglobin Yhb1 in yeast), respiratory complex II, respiratory complex III, and yeast-specific proteins (respiratory complex IV assembly factor Mss51, cytochrome *c* peroxidase Ccp1, and *L*-lactate cytochrome *c* oxidoreductase Cyb2) (reviewed in [7]). To date, the mechanism of heme *b* translocation to these proteins is unknown.

To form the covalently bound *c*-type heme found in cytochrome *c* (human CYCS or yeast paralogs Cyc1/Cyc7) and the respiratory complex III subunit cytochrome *c*_1_ (human CYC1 or yeast Cyt1), heme *b* is covalently bound to the hemoprotein via thioether bonds with highly conserved cysteine residues [6,289,290]. The process by which heme *b* is transported to these heme-*c*-containing proteins is partially understood. The mammalian holocytochrome *c* synthase (HCCS) and the yeast cytochrome *c* heme lyases (Cyc3 and Cyt2) are responsible for directly delivering and covalently attaching heme *b* to cysteines in cytochrome *c* and cytochrome *c*_1_. However, the factors that mediate heme *b* transfer to HCCS, Cyc3, and Cyt2 remain elusive [289,291,292].

In the heme *a* modification pathway, heme *b* is extensively modified via the addition of a hydroxyfarnesyl group to one of its vinyl carbons by heme *o* synthase (COX10) and the conversion of a methyl group to an aldehyde by heme *a* synthase (COX15). Heme *a* is exclusively found in the COX1 subunit of respiratory complex IV, and heme chaperones for delivering and installing the cofactor have not been identified. The complex IV assembly factors SURF1 (Shy1 in yeast) and Coa2 have been posited as heme chaperones for this pathway, but each protein appears to only be involved in the hemylation of COX1 rather than trafficking heme to the site of complex assembly [293,294,295,296,297,298,299,300,301,302].

## 4. Iron Export from Mitochondria

The role of mitochondria in iron homeostasis is not limited to utilization in ISC biogenesis and heme synthesis; the labile iron pool in mitochondria can also act as an iron depot to supply the rest of the cell. This iron is in the form of “free” iron (a term for iron ions with various low-molecular-mass ligands), heme iron, and ISC intermediates.

**Figure 3:**
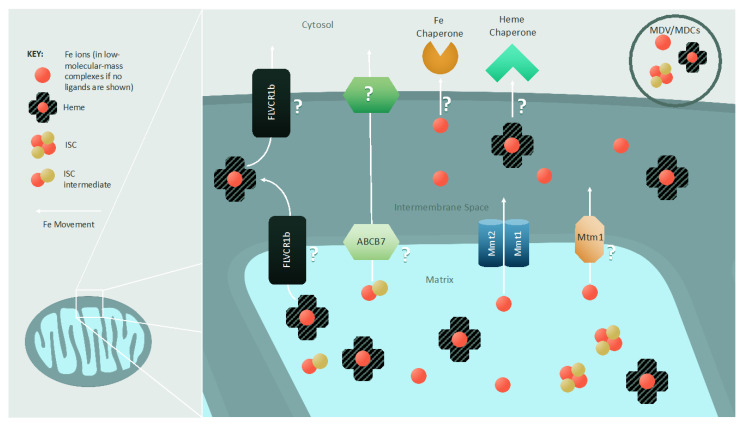
Export of mitochondrial iron and iron-containing cofactors: Iron is exported from mitochondria as heme or metal ions in low-molecular-mass complexes. Additionally, a presently unidentified intermediate required for cytosolic ISC biogenesis is also exported from mitochondria. In yeast, three inner mitochondrial membrane iron exporters have been identified: Mmt1, Mmt2, and Mtm1. In the case of heme export, FLVCR1b is postulated to play a role, but it is unclear where FLVCR1b localizes within the mitochondrion or to what extent it facilitates heme export. The ISC intermediate necessary for cytosolic ISC biogenesis is trafficked across the inner mitochondrial membrane by Atm1 in yeast and ABCB7 in mammals. Methods of export across the outer mitochondrial membrane remain unknown. It is hypothesized that various protein channels allow for the flow of these iron species, and that iron and cofactor chaperones directly accept or extract their cargo from the mitochondrion. A non-protein-based mechanism for iron, heme, and ISC intermediate export is also possible via mitochondria-derived vesicles (MDVs) and compartments (MDCs).

Several proteins have been hypothesized to export “free” iron (Figure 3), with most studies carried out in *S. cerevisiae*. The mitochondrial metal transporters Mmt1 and Mmt2 conserved in fungi and plants have been identified as exporters of mitochondrial iron [303,304]; their overexpression results in cytosolic iron accumulation, while their deletion decreases cytosolic iron load [304]. Another protein postulated to be involved in mitochondrial iron export in yeast is Mtm1. Deletion of Mtm1 in *S. cerevisiae* results in mitochondrial iron accumulation and enhanced cellular iron uptake, while also inducing the misincorporation of iron into the mitochondrial manganese-containing superoxide dismutase [305,306,307]. The vertebrate functional ortholog of Mtm1 (Slc25a39) is required for iron’s integration into protoporphyrin IX to produce heme *b* in murine erythroid cells; its deletion induces anemia in zebrafish, cementing its role in mitochondrial iron homeostasis, while suggesting that further studies are needed to completely elucidate the role of Mtm1-related proteins [308]. As discussed earlier, a sulfur- (and possibly iron-) containing intermediate is exported from the mitochondrion to enable cytosolic ISC biogenesis, and its export may involve proteins such as Atm1 and Mdl1 (in yeast) and ABCB7 and ABCB8 (in mammals) [270,271,272,273,274,275].

Non-protein pathways—specifically, mitochondria-derived compartments (MDCs) and mitochondria-derived vesicles (MDVs)—are also hypothesized to participate in the efflux of “free” iron, along with ISCs and heme. MDCs and MDVs are single- and double-membrane-bound vesicles with an approximate diameter of 70–100 nm that can interact with other subcellular compartments, such as the ER, lysosome, and peroxisome [309,310,311,312,313,314]. MDVs have been shown to contain various cargo loads, including metabolites and proteins [312,315], and proteomic analysis of purified MDVs has uncovered high levels of mitochondrial ISC biogenesis proteins and proteins capable of binding iron [316]. The strategy of mobilizing “free” iron, ISCs, and heme through MDCs and MDVs would be advantageous in terms of minimizing their cytotoxic effects and enhancing their availability.

The mechanism of heme export from mitochondria remains elusive and debated, but several factors have been implicated. To date, only one protein—a mitochondrial isoform of FLVCR protein 1b—has been identified as a potential heme exporter. Erythropoiesis is terminated and heme is accumulated in mitochondria following FLVCR1b depletion, while overexpression of this transporter increases cytosolic heme levels. FLVCR1b lacks a defined heme-binding/transport segment akin to that found in its isozyme FLVCR1a [317,318]. Significantly, FLVCR1a and FLVCR1b appear to be specific to erythroid cells. Moreover, they are not found in lower eukaryotes, suggesting that other, more conserved mechanisms of heme export should exist.

Once heme is trafficked to the mitochondrial outer membrane, a cytosolic chaperone is required to accept heme from the mitochondrion. Progesterone receptor membrane component 1 (PGRMC1) and heme-binding protein 1 (HBP-1) are candidates for this function, but further studies are required to delineate their role in heme extraction from mitochondria.

Some of the iron exported from mitochondria may ultimately be exported from the cell. Mechanisms of cellular iron export are poorly understood; however, ferroportin has been identified as an exporter of non-heme iron, and multidrug resistance protein 5 (MRP-5) and FLVCR1a both export heme [10,319,320,321,322]. In macrophages, heme-responsive gene 1 (HRG-1), which is homologous to a heme plasma membrane transporter in *Caenorhabditis elegans*, exports heme from the phagolysosome to the cytosol during erythrophagocytosis [323,324].

## 5. Concluding Remarks

Iron is essential for cellular physiology and life. Since this metal obtained from the environment is redox-active, cellular iron import and transport are highly regulated processes. Much is known about how iron is trafficked extracellularly, as well as cytosolically, and how it is delivered to various organelles, but there remain many gaps in the information known about mitochondrial iron transport and the movement of iron-containing cofactors. One currently elusive topic that requires further elucidation is the coordination of iron’s delivery to mitochondria and transport across the OMM and IMM, as well as how those pathways cooperate to maintain cellular iron homeostasis. Regarding IMM transport, mechanistic studies of both yeast and mammalian mitoferrins will provide insight into how iron import is mediated and regulated at the molecular level.

Even though a breadth of knowledge has been generated regarding the trafficking of iron to the mitochondrial ISC biogenesis machinery and the transport of the sulfur- (and possibly iron-) containing intermediate to the cytosol for ISC biogenesis via the CIA, many outstanding questions about this process remain. For example, a defined role of frataxin has not been delineated, despite extensive analysis of this protein. If frataxin does not directly provide iron to the ISC biogenesis scaffold, then the involvement of the mitochondrial labile iron pool or additional factors would need to be identified. The molecular mechanism for transporting the sulfur-containing intermediate to the cytosol and its precise identity remain elusive. Unraveling the roles of ABCB7 and ABCB8 in mitochondrial iron homeostasis, and whether these two proteins mediate the transfer of this intermediate, could lead to therapies for diseases where mitochondrial iron homeostasis is implicated. Determining the mechanism of ISC transfer from the ISC biogenesis scaffold to apoproteins is another important goal. The LYR motif being commonly found in some, but not all, ISC proteins suggests that the mechanisms of substrate recognition by the HSPA9–HSC20 system and GLRX5 for the delivery of 2Fe-2S clusters to target proteins require further elucidation. Likewise, the molecular mechanisms involved in delivery of 4Fe-4S clusters by specialized ISC trafficking proteins are still under study. The mechanism by which the sideroflexins support iron homeostasis and ISC biogenesis will also be enlightening, and may answer some of the above questions.

Further research is necessary to determine the factors involved in mitochondrial heme trafficking and export. Similarly, limited information is known about how heme *b*, heme *c*, and heme *a* are trafficked to their sites of utilization within mitochondria. Defining these processes and mechanisms will provide answers to a plethora of fundamental biological questions, and contribute to a more expansive understanding of mitochondrial iron homeostasis and mitochondrial iron-related disease pathologies, which have been extensively reviewed [1,9,10,325] Finally, it will be important to place many of these fundamental mechanisms into physiological context by addressing their tissue and/or organ specificity, which may hold clues to understanding the molecular bases of iron management-related disorders in humans.

## Figures and Tables

**Table 1:** Conservation of proteins involved in maintaining mitochondrial iron homeostasis. (SA = sideroblastic anemia; MMDS = multiple mitochondrial dysfunctions syndrome).

Pathway	*S. cerevisiae*	Mammalian	Function	Pathology
Mitochondrial Fe Import	Por1/Por2	VDAC	Mitochondrial Fe import?	-
	-	DMT1	Mitochondrial Fe import	Hypochromic microcytic anemia [70,71,72]
	-	PCBP2	Fe transporter	-
	Mrs3/Mrs4	MFRN1/ MFRN2	IMM Fe importer	-
	Rim2	-	IMM low-affinity Fe importer	-
	Mdl1 (peptide transporter)	ABCB10	Stabilization of MFRN1/IMM biliverdin transporter in mammals?	-
	-	MCU	IMM Ca (Fe?) import	-
	-	SFXN1-5	Similar and differing functions in serine transport and Fe transport across IMM	Mitochondriopathy; macrocytic anemia [73]
Mitochondrial Iron Storage	-	FTMT	Mitochondrial Fe storage	-
ISC Biogenesis and Maturation of ISC Proteins	Nfs1	NFS1	Cysteine desulfurase/sulfur donation	Combined oxidative phosphorylation deficiency [74,75]
	Isd11	ISD11	Nfs1 stabilization/regulation	Combined oxidative phosphorylation deficiency [76]
	Acp1	ACP1	Nfs1 stabilization/regulation	-
	Yfh1	FXN	Iron donation?/ISC biogenesis regulator	Friedreich’s ataxia [77,78,79,80,81,82,83]
	Isu1/Isu2	ISCU	Core ISC biogenesis scaffold	Hereditary myopathy with lactic acidosis [84,85,86,87]
	Yah1	FDX2	Electrons for ISC synthesis	Episodic mitochondrial myopathy [88,89]
	Arh1	FDXR	Electrons for ISC synthesis	Auditory neuropathy; optic atrophy [90]
	Ssq1	HSPA9	ISC transfer	SA [91,92]
	Jac1	HSC20	ISC transfer	-
	Mge1	GRPEL1	Nucleotide release factor for Ssq1/HSPA9	-
	Grx5	GLRX5	ISC transfer	SA [93,94,95]
	Isa1	ISCA1	4Fe-4S synthesis	MMDS [96,97]
	Isa2	ISCA2	4Fe-4S synthesis	MMDS [98,99,100,101]
	Iba57	IBA57	4Fe-4S synthesis	MMDS [102,103,104,105,106,107]
	Nfu1	NFU1	Maturation of ISC proteins	MMDS [108,109]
	-	NUBPL	Maturation of ISC proteins	Mitochondrial complex I deficiency [110,111,112,113]
	Bol3	BOLA3	Maturation of ISC proteins	MMDS 2 [108,114,115]
	Bol1	BOLA1	Maturation of ISC proteins	-
	Atm1	ABCB7	Fe or ISC intermediate export	SA with ataxia [116,117,118]
	-	ABCB8	Fe or ISC intermediate export?	-
Heme Biosynthesis and Modification	Hem15	FECH	Final step in formation of heme *b*	Erythropoietic protoporphyria [119,120,121,122,123,124,125]
	Cox10	COX10	Formation of heme *o* intermediate from heme *b*	Mitochondrial complex IV deficiency [126,127,128]
	Cox15	COX15	Formation of heme *a* from heme *o* intermediate	Mitochondrial complex IV deficiency [129,130,131,132]
	Cyc3/Cyt2	HCCS	Heme installation in cytochrome *c* and cytochrome *c*_1_	-
Mitochondrial Fe Export	Mmt1/Mmt2	-	Mitochondrial Fe export	-
	Mtm1	Slc25a39	IMM Fe export	-
	-	FLVCR1b	Mitochondrial heme export?	-
	Dap1	PGRMC1	Cytosolic acceptor of mitochondrial heme?	-
	-	HBP-1	Cytosolic acceptor of mitochondrial heme?	-

## Data Availability

Not applicable.
